# Pediatric Headache: A Practical Approach to Clinical Diagnosis and Management

**DOI:** 10.7759/cureus.116072

**Published:** 2026-09-10

**Authors:** Nishat Ahmed

**Affiliations:** 1 Neurology, Sardar Patel Medical College, Bikaner, IND

**Keywords:** cgrp antagonists, cgrp mabs, cluster headache, pediatric headache, pediatric migraine, preventive treatment, red flags, secondary headache, tension-type headache, trigeminal autonomic cephalalgias

## Abstract

Headache is among the most common neurological complaints of childhood and adolescence and can substantially impair school attendance and quality of life. A practical approach to the child with headache rests on four tasks: recognizing the primary headache phenotype, screening for the minority of children whose headache is secondary to another disorder, assessing how much the headache is disabling the child, and matching treatment intensity to that disability. Migraine and tension-type headache account for most primary headache presentations, but the trigeminal autonomic cephalalgias and, rarely, painful cranial neuropathies must also be recognized because their management differs fundamentally from that of migraine. Diagnosis is clinical, based on a structured history, assessment of disability, and a careful general and neurological examination; red flags should prompt reassessment and risk stratification rather than reflexive neuroimaging, and in a child with a typical primary headache phenotype and a normal examination, routine imaging, laboratory testing, or electroencephalography is generally unnecessary. Management is multimodal: education and lifestyle measures form the foundation of care; acute treatment uses evidence-based, age-appropriate analgesics and triptans; and preventive treatment is reserved for children with substantial headache-related disability, bearing in mind the unusually high placebo response documented in pediatric migraine prevention trials. Several calcitonin gene-related peptide-targeted and device-based therapies have emerged for pediatric migraine since 2024, though pediatric evidence and regulatory status vary substantially by therapy. Tension-type headache and the trigeminal autonomic cephalalgias require separate diagnostic and therapeutic consideration, and treatment of the latter reflects evidence largely extrapolated from adults and case series, rather than controlled pediatric trials. This review provides a practical, evidence-based framework for the clinical diagnosis and management of pediatric headache for the general clinician.

## Introduction and background

Headache is one of the most common neurological complaints of childhood and adolescence. Globally, an estimated 58-60% of children and adolescents report experiencing headache within a defined period, such as the preceding year, encompassing both primary and secondary headache disorders; migraine affects approximately 7.7-9.1% of this population [1]. Headache is also a common disease-related cause of school absenteeism in children, and children with migraine have impaired academic performance and quality of life comparable to that of children with rheumatoid arthritis or cancer [1].

Pediatric headache is not a single entity, and it is not simply small-adult migraine. Migraine and tension-type headache (TTH) together account for the great majority of primary headache presentations, but the trigeminal autonomic cephalalgias (TACs) - cluster headache, paroxysmal hemicrania, the short-lasting unilateral neuralgiform headache attacks with conjunctival injection and tearing (SUNCT) or with cranial autonomic symptoms (SUNA), and hemicrania continua - and, rarely, painful cranial neuropathies such as trigeminal or occipital neuralgia, must be recognized because their treatment differs fundamentally from that of migraine [2-4]. Clinical phenotype also varies with age: younger children more often have bilateral, shorter-duration pain and prominent gastrointestinal or behavioral manifestations, while older children and adolescents present in a manner closer to the adult phenotype [1].

The central clinical task in every child presenting with headache is therefore fourfold: to recognize the specific primary headache phenotype; to identify the minority of children whose headache is a symptom of an underlying secondary disorder; to assess how much the headache is actually disabling the child, rather than relying on frequency alone; and to select treatment in a stepwise way that matches that disability. Most children with recurrent headache can be diagnosed clinically on the basis of a structured history and examination; an atypical or progressive pattern, or an abnormal neurological examination, should prompt reconsideration of the diagnosis and selective investigation [1,5].

Pediatric headache has been the subject of recent comprehensive reviews [6], and this article does not claim to be the first to cover the topic. What it aims to add is a practical, phenotype-based clinical framework that brings together current evidence - including the calcitonin gene-related peptide (CGRP)-targeted, device-based, and other therapies that have emerged since 2024 - with an explicit account of how strong that evidence actually is, so that a general pediatrician or neurologist can use it directly in clinic rather than only as background reading. The objective of this review is to provide a practical, evidence-based framework for the clinical diagnosis and management of pediatric headache. This review provides a practical, source-based approach to the classification, clinical assessment, red-flag recognition, investigation, and management of pediatric headache, covering migraine, tension-type headache, the trigeminal autonomic cephalalgias, and painful cranial neuropathies, intended for pediatricians, physicians, and neurologists managing children with headache in general clinical practice [6].

Search strategy and selection criteria

The literature for this review was identified through a structured, topic-based search of PubMed, supplemented by other web-based sources, rather than a systematic review process. Literature was searched up to mid-2026, without formal restriction by publication date. Representative search terms included pediatric headache, pediatric migraine, tension-type headache, trigeminal autonomic cephalalgias, cluster headache, CGRP monoclonal antibodies, gepants, fremanezumab, and remote electrical neuromodulation, combined with terms specific to each clinical domain reviewed. The reference list accordingly includes both foundational works published before 2020 - including the International Classification of Headache Disorders, third edition (ICHD-3) classification and landmark pediatric migraine practice guidelines and trials - alongside more recent literature, with the majority of references published from 2020 onward, including several from 2025-2026 reflecting current evidence. A dedicated project folder for pediatric headache was created, and the topic was divided into clinically relevant domains, including clinical phenotype and diagnosis, migraine, tension-type headache, the trigeminal autonomic cephalalgias, acute and preventive pharmacological treatment, CGRP-targeted therapies and gepants, investigations, and post-traumatic headache. Approximately 30 articles were initially identified and organized by topic in a reference-management library, of which 25 were ultimately included in the narrative synthesis presented in this manuscript. Each domain was reviewed and synthesized separately, and the resulting sections were combined into this manuscript. This is a narrative, not a systematic, review: no formal database search strings, independent screening, or risk-of-bias assessment were used.

## Review

Classification of pediatric headache

The International Classification of Headache Disorders, third edition (ICHD-3), remains the standard diagnostic framework. It separates primary headache disorders from secondary headaches and from painful cranial neuropathies and other facial pain. Within the primary headache group, the major categories relevant to children are migraine, tension-type headache, the trigeminal autonomic cephalalgias, and other primary headache disorders such as primary stabbing headache and new daily persistent headache [2]. Classification is clinically useful because it forces an early distinction between a primary headache phenotype and a headache attributable to an underlying disorder, and because specific diagnoses guide specific treatment [1]. Table 1 summarizes the clinical phenotype, typical duration and frequency, key associated features, and diagnostic clue for each disorder, together with its practical relevance.

**Table 1:** Classification and clinical phenotypes of pediatric headache Source: [1-4] N/V - nausea and/or vomiting; NDPH - new daily persistent headache; SUNCT - short-lasting unilateral neuralgiform headache attacks with conjunctival injection and tearing; SUNA - short-lasting unilateral neuralgiform headache attacks with cranial autonomic symptoms

Disorder	Phenotype	Duration/frequency	Key features	Diagnostic clue
Migraine without aura	Uni/bilateral, pulsating, mod-severe	2h-72h; ≥5 attacks	N/V, photo/phonophobia	Recurrent stereotyped attacks
Migraine with aura	Uni/bilateral, pulsating, mod-severe; preceded/accompanied by aura	2h-72h headache; ≥2 attacks with aura	Fully reversible visual, sensory, or speech/language aura; N/V, photo/phonophobia in minority	Recurrent attacks with reversible aura
Tension-type headache	Bilateral, pressing, mild-mod	30 min-7d	≤1 of photo/phonophobia; no N/V	Featureless bilateral pressure
Cluster headache	Unilateral, orbital/temporal, severe	15 min-180 min; 1-8/d	Ipsilateral autonomic signs, restlessness	Autonomic signs + circadian pattern
Paroxysmal hemicrania	Unilateral, orbital/temporal	2 min-30 min; ≥5/d	Autonomic features	Resolves with indomethacin
SUNCT/SUNA	Unilateral, trigeminal	5s-240s; 3-200/d	Conjunctival injection/tearing (SUNCT)	Very brief, very frequent
Hemicrania continua	Unilateral, continuous	Continuous, >3 mo	Autonomic features inconsistent	Resolves with indomethacin
Other primary (stabbing; NDPH)	Variable/diffuse	Seconds; or continuous >3 mo	No autonomic features	Brief stabs / abrupt persistent onset
Painful cranial neuropathies	Unilateral, paroxysmal	Brief, stimulus-triggered	Nerve-distribution pain	Imaging to exclude structural cause
Secondary headache	Variable, atypical	Variable	Systemic/neurologic red flags	Treat underlying cause

Clinical assessment and diagnosis

Age and developmental considerations

Headache phenotype changes across childhood and adolescence, and history-taking must be adapted accordingly. In young children, migraine attacks may be brief - ICHD-3 allows a minimum attack duration of two hours in children under 18 years, though some experts have proposed a lower threshold in very young children - and behavioral change (stopping play, seeking a dark or quiet room, lying down, becoming pale and quiet) may communicate photophobia, phonophobia, or fatigue more reliably than direct questioning, since drawings and behavior, not verbal report, often carry the diagnostic clue in this age group [1]. Bilateral pain is common in children (more than 80% report bilateral headache), with unilateral localization becoming more characteristic in later adolescence; gastrointestinal symptoms such as vomiting and pallor and behavioral withdrawal are prominent in young children, while cranial autonomic symptoms accompanying migraine are usually bilateral in children and should raise the possibility of a TAC only when clearly unilateral [1]. In adolescents, menstrual association, sleep restriction, school stress, mood symptoms, and medication-overuse patterns deserve specific and direct questioning, and adolescents should ideally be interviewed both with and without a parent or guardian present [1].

Every child with headache should undergo a general and neurological examination, including blood pressure, growth parameters, and head circumference. The neurological examination should assess mental status, cranial nerves (including a fundoscopic examination for papilledema), motor and sensory function, coordination, and gait, together with palpation of the sinuses, examination of the neck and spine, and assessment of the temporomandibular joint [1]. An abnormal neurological examination substantially changes the subsequent diagnostic pathway and lowers the threshold for investigation.

History-taking should systematically establish onset (age, sudden versus gradual, first-or-worst headache), temporal pattern (frequency, duration, progression), pain phenotype (location, quality, severity, aggravation by activity), associated symptoms (nausea, photophobia/phonophobia, cranial autonomic features), aura or other neurological symptoms, triggers and context (sleep, meals, hydration, exercise, stress, menstruation), acute-medication use and frequency, functional impact (school absence, academic and social impairment), and relevant comorbidity or family history [1]. Headache frequency alone does not adequately describe disease burden. The Pediatric Migraine Disability Assessment (PedMIDAS) is a validated six-item, self-administered instrument for quantifying migraine-related disability over the preceding three months, and a headache diary can complement this assessment when frequency, triggers, menstrual association, medication use, or treatment response is uncertain [1]. Taken together, a structured history and a validated disability measure tell the clinician not only what type of headache this is, but how much it is affecting the child - and it is this second question, as much as the diagnosis itself, that determines how intensively the headache should be treated.

Primary headache disorders

Migraine

Migraine without aura requires, under ICHD-3, at least five attacks lasting 2-72 hours in children and adolescents younger than 18, with at least two of unilateral location, pulsating quality, moderate-to-severe intensity, or aggravation by routine physical activity, together with nausea/vomiting or both photophobia and phonophobia [2]. Migraine with aura consists of recurrent, fully reversible neurological symptoms - most often visual, but also sensory or speech/language - that typically develop gradually before or with the headache; the history should establish the sequence, gradual evolution, and reversibility of these symptoms, because several secondary disorders can mimic aura [2]. Chronic migraine is defined as headache on at least 15 days per month for at least three months, with at least eight days meeting migraine criteria; it is more common in girls, and its prevalence rises through adolescence [1]. Disability from pediatric migraine can be substantial: nearly one in 10 children have recurrent headache due to migraine, and disability is comparable to that seen with other major chronic pediatric illnesses [1].

Tension-type headache

Tension-type headache is the most prevalent primary headache worldwide and occurs in an estimated 10-24% of children and adolescents, though pooled prevalence estimates vary widely (0.9-72.3%) across heterogeneous pediatric studies [7]. ICHD-3 divides TTH into infrequent episodic (<1 day/month), frequent episodic (1-14 days/month for >3 months), and chronic (≥15 days/month for >3 months) subtypes, each requiring at least two of bilateral location, pressing/non-pulsating quality, mild-to-moderate intensity, and lack of aggravation by routine activity, together with the absence (episodic) or near-absence (chronic) of nausea, photophobia, or phonophobia [7]. A typical episode lasts four to six hours, though attacks can range from 30 minutes to several days [7].

Diagnostic overlap between TTH and migraine is common in children: many children with TTH report migrainous features such as unilateral pain, throbbing quality, or mild photophobia/phonophobia, while migraine in children is more often bilateral and shorter in duration than in adults, which further blurs the distinction [7]. Roughly a quarter of children with migraine are reported to switch to a TTH phenotype over long-term follow-up, and vice versa, indicating that TTH and migraine may in part represent a developmental and phenotypic spectrum rather than two wholly separate entities [7]. Psychosocial factors are relevant to both the presentation and course of TTH: adverse childhood experiences, family psychiatric history, and dysfunctional family relationships have been associated with headache persistence and with mood and anxiety comorbidity, and temporomandibular joint dysfunction, bruxism, and psychosocial stressors should specifically be sought as contributors or mimics [7].

Management begins with lifestyle measures - regular sleep, hydration, consistent meals, limited caffeine, regular physical activity, and limitation of screen time - which constitute first-line treatment [7]. Behavioral approaches (cognitive-behavioral therapy (CBT), relaxation training, and biofeedback, particularly electromyographic and thermal biofeedback) are second-line and are well tolerated, with pediatric studies showing greater biofeedback response than in adults [7]. Pharmacological treatment is reserved for cases not responding to lifestyle and behavioral measures, and pediatric evidence for prophylactic pharmacotherapy in TTH specifically is very limited; no drug is approved specifically for this indication in children, and use is off-label. Where used, amitriptyline for TTH prophylaxis reflects evidence largely extrapolated from adults and small pediatric series, rather than randomized pediatric TTH trials, and should not be presented as established pediatric evidence [7].

Trigeminal autonomic cephalalgias

The TACs are unified by strictly unilateral, severe headache with ipsilateral cranial autonomic features and, in most, a sense of restlessness or agitation; they are differentiated from one another chiefly by attack duration, frequency, and response to indomethacin [2,3]. They are rare in children, and pediatric treatment recommendations for all TACs reflect evidence largely extrapolated from adults, supplemented by pediatric case series, rather than controlled pediatric trials [3,4].

Cluster headache: Cluster headache is diagnosed using the same ICHD-3 criteria in children as in adults: at least five attacks of severe unilateral orbital, supraorbital, and/or temporal pain lasting 15-180 minutes untreated, with either at least one ipsilateral cranial autonomic sign or a sense of restlessness/agitation, occurring with a frequency of one every other day to eight per day [2,3]. Pediatric prevalence is estimated at 0.03-0.5% [3,4], with most pediatric cases beginning in adolescence after age 15, although onset as early as infancy has been reported [3]. As in adults, there is a male predominance overall, but this is less clear before puberty, when the sex ratio approaches 1:1 [3]. Compared with adults, children show a lower attack frequency (typically 1-6/day), less prominent restlessness and autonomic signs, but more frequent nausea, vomiting, photophobia, and phonophobia - features that predispose to misdiagnosis as migraine [3]. Diagnostic delay is substantial, averaging roughly two to three years across case-series and systematic-review data [3,4]. Because pediatric cluster headache is rare and its mimics (including intracranial neoplasm, most often in the pituitary or cavernous region, and vascular lesions) are important, brain magnetic resonance imaging (MRI) with vascular imaging is routinely recommended at diagnosis even when the clinical picture is otherwise typical [3]. Migraine without aura is the most important differential; unlike migraine, cluster headache pain typically drives restlessness rather than a preference to lie still, and autonomic signs in cluster headache are unilateral (ipsilateral to pain) rather than the bilateral autonomic signs more typical of migraine [3].

Paroxysmal hemicrania: Overlaps with cluster headache in both duration (2-30 minutes) and frequency (>5/day) but is distinguished by a dramatic, near-diagnostic response to indomethacin, whereas cluster headache is traditionally refractory to indomethacin [3].

SUNCT/SUNA: Characterized by very brief attacks (5-240 seconds) at very high frequency (3-200/day); SUNCT is distinguished from SUNA by the presence of conjunctival injection and tearing, whereas SUNA may present with only one or no autonomic feature [3].

Hemicrania continua: A continuous, strictly unilateral headache with exacerbations, present for more than three months, with an absolute response to indomethacin required for diagnosis; autonomic features are not universal, so an indomethacin trial should be considered in any child with continuous side-locked headache [1,3].

Other primary headaches

Primary stabbing headache consists of very brief (seconds), often multiple daily stabs of pain without autonomic accompaniment, most often occurring in children who also have another primary headache disorder such as migraine; it is usually alarming but benign and rarely requires specific treatment [1,3]. New daily persistent headache describes an abrupt, clearly remembered onset of headache that becomes daily and persists beyond three months in a child without a prior significant headache history, and is diagnosed only after appropriate exclusion of secondary causes [1].

Painful cranial neuropathies are rare in children and, when they occur, warrant particular caution regarding an underlying secondary cause.

Trigeminal neuralgia presents with unilateral pain confined to the distribution of one or more branches of the trigeminal nerve, sometimes with mild ipsilateral autonomic accompaniments, and is distinguished from cluster headache by its typically much shorter attack duration (usually under two minutes), lack of circadian or circannual periodicity, and precipitation by innocuous stimuli (light touch, chewing, brushing teeth) within the affected trigeminal distribution [2,3]. Trigeminal neuralgia in pediatric age is extremely rare, and the small number of reported cases have predominantly had a secondary etiology, most often vascular; a child presenting with a trigeminal-neuralgia phenotype therefore warrants imaging directed at excluding a structural or vascular cause rather than a primary diagnosis [3].

Occipital neuralgia presents with paroxysmal, shooting or stabbing pain in the distribution of the greater or lesser occipital nerve, sometimes with tenderness over the nerve on palpation; it is likewise uncommon in children, and a secondary cause (including craniocervical structural pathology) should be actively considered, particularly when tenderness, sensory change, or a clear structural trigger is present.

These entities are noted here for completeness because they are important, if rare, differentials for unilateral, paroxysmal pediatric facial or head pain; specific pediatric prevalence or treatment data are too limited to support firm management recommendations beyond referral for specialist assessment and, where appropriate, neuroimaging directed at excluding a secondary cause.

Red flags and secondary headache

The presence of a red flag should prompt reassessment of the diagnosis and clinical context - a trigger for risk stratification, not an automatic indication for neuroimaging [1,5,8]. A useful pediatric framework is the mnemonic SNOOP4Y: Systemic signs/symptoms, Neurologic signs/symptoms, Onset sudden, Onset in sleep/early morning, Positional exacerbation/Precipitated by Valsalva, (lack of family history in) Parents, Progressive or new, and Young age [1,8]. A single nonspecific feature - early-morning headache, vomiting, or occipital location - has limited predictive value in isolation and should not by itself trigger imaging; conversely, papilledema, a focal neurological deficit, altered consciousness, a new seizure, thunderclap onset, or a clearly progressive pattern should substantially lower the threshold for urgent evaluation (Table 2) [1,5]. Life-threatening secondary causes such as brain tumors are found in only approximately 2-3% of children presenting to the emergency department with headache, and in about 1% of children presenting to primary care, underscoring that most childhood headache is primary [1].

**Table 2:** Red flags in pediatric headache and suggested evaluation Source: [1,5,8] MRI - magnetic resonance imaging; ICP - intracranial pressure; MRV - magnetic resonance venography; CTV - computed tomography venography; LP - lumbar puncture; EEG - electroencephalography; CT - computed tomography; TAC - trigeminal autonomic cephalalgia; CNS - central nervous system

Red flag	Suggested step
Typical phenotype, normal exam	No routine imaging/labs/EEG
Systemic illness (fever, weight loss, malignancy)	Targeted labs; consider infectious/oncologic cause
Abnormal neuro exam (focal deficit, papilledema, seizure)	MRI brain
Thunderclap onset	Urgent workup; consider vascular emergency
Positional (worse upright/supine)	MRI; consider hypotension or raised ICP
Valsalva/cough-triggered	MRI; consider posterior fossa pathology
Suspected venous thrombosis	MRV/CTV
Suspected CNS infection/↑ICP	LP after imaging
Suspected seizure	EEG
New TAC/cluster phenotype	MRI with vascular imaging
Trigeminal-neuralgia phenotype	Imaging to exclude structural cause
Significant head trauma	CT if indicated
Progressive/atypical pattern	Reassess diagnosis; lower threshold to investigate

Investigations and neuroimaging

As already noted, a child with a typical primary headache phenotype, a normal examination, and no red flags needs no routine laboratory testing, EEG, or neuroimaging; this is supported by both older pediatric practice-parameter guidance and by more recent studies confirming that neuroimaging is unnecessary for children with stable headache frequency, no red flags, and a normal examination [1,5,9-12]. The practical value of this restraint is that selective, clinically directed investigation - rather than a blanket headache work-up - reduces unnecessary radiation exposure, sedation requirements, and incidental findings without missing the children who genuinely require imaging [1,5].

When investigation is indicated (Table 2), MRI is the preferred non-emergency modality for suspected structural or inflammatory pathology, since it avoids ionizing radiation; CT is reserved for acute neurological emergencies, rapid assessment, or selected trauma/hemorrhage situations where MRI is not immediately feasible; magnetic resonance or computed tomography venography is used specifically when cerebral venous sinus thrombosis is suspected, since routine MRI or CT does not reliably exclude it; lumbar puncture is reserved for selected infection, intracranial-pressure, or subarachnoid-hemorrhage pathways after appropriate imaging; EEG is reserved for suspected seizures and does not diagnose migraine; and laboratory testing should be directed at a specific suspected systemic, inflammatory, infectious, or metabolic cause rather than obtained routinely [1,5]. Cluster headache and other TAC presentations are a specific exception to a purely clinical approach: because these disorders are rare in children and their mimics (particularly pituitary and cavernous-region neoplasms and vascular lesions) are clinically important, MRI with vascular imaging is generally recommended at first diagnosis even when the clinical picture appears typical [3]. Similarly, a trigeminal-neuralgia phenotype in a child should prompt imaging to exclude a secondary vascular or structural cause, given how rarely trigeminal neuralgia is primary at this age [3].

Management

Education and lifestyle measures

Education and lifestyle optimization form the foundation of care for every primary headache disorder. Consistent, age-appropriate sleep; regular meals and adequate hydration; regular physical activity; and identification of individual (rather than presumed universal) triggers should be discussed with every family. Families should be counseled that broad, unindividualized dietary restriction is not well supported, and that consistent lifestyle habits reduce headache burden more reliably than eliminating specific presumed triggers [1]. Behavioral interventions such as cognitive-behavioral therapy, biofeedback, and relaxation training, delivered alongside or in place of pharmacological measures, have evidence supporting reduced attack frequency and disability across pediatric headache disorders [13]. A headache diary is a useful adjunct for tracking frequency, triggers, disability, acute-medication use, and treatment response across all headache types.

Acute treatment of pediatric migraine

Acute treatment should be initiated early, in an adequate dose, using a formulation suited to the child's age and attack characteristics, with the explicit goals of rapid, sustained relief and return of function [1,5].

Route selection should be individualized: oral therapy is reasonable when nausea is limited, while intranasal triptan formulations are preferred when nausea, vomiting, or rapid escalation of pain limits reliable oral absorption [1,5]. For adolescents with an incomplete response to a triptan alone, adding an nonsteroidal anti-inflammatory drug (NSAID; ibuprofen or naproxen) is guideline-supported, with sumatriptan/naproxen the most established pediatric combination [5,14]. Treatment response should be judged by both pain relief and restoration of function, and every acute-treatment plan should specify what to do if the first dose fails, rather than encouraging repeated unscheduled dosing [1].

Medication-overuse counseling should begin at the time acute treatment is first prescribed, well before a child approaches the frequency of use that risks medication-overuse headache (discussed further below); as a practical guide, the 2019 American Academy of Neurology/American Headache Society (AAN/AHS) acute-treatment guideline counsels limiting simple analgesics to 14 or fewer days per month and triptans to nine or fewer days per month [5]. Opioids have no established role in acute pediatric migraine treatment and should be avoided [1,5]. Triptans are contraindicated in children with ischemic cardiovascular or cerebrovascular disease and accessory cardiac conduction pathways such as Wolff-Parkinson-White syndrome; the traditional contraindication in hemiplegic migraine and migraine with brainstem aura is now considered outdated by some authorities, but specialist referral is reasonable for these uncommon phenotypes [1,5].

Preventive treatment of pediatric migraine

Preventive treatment should be considered when migraine is frequent, prolonged, or disabling; substantially interferes with school or daily functioning; or when acute treatment is ineffective, poorly tolerated, contraindicated, or being used too frequently [1]. Most pediatric prevention trials have used a minimum of four headache days per month and/or three to four migraine attacks per month as an inclusion threshold, together with significant migraine-related disability (PedMIDAS >30) [1].

A defining feature of pediatric migraine prevention trials is an unusually high placebo response: 30-61% of children receiving placebo achieved at least a 50% reduction in headache frequency across the trials reviewed in the 2019 AAN/AHS prevention guideline [1,15]. This was demonstrated most starkly by the CHAMP trial, in which approximately half to two-thirds of children in all three arms (topiramate, amitriptyline, and placebo) achieved a 50% or greater reduction in headache days, with no significant difference between either active drug and placebo - a result better understood as showing that structured, multi-component care (diagnosis, evidence-based acute treatment, lifestyle counseling, and avoidance of medication overuse) benefits most children, rather than as evidence that pharmacological prevention does not work [16]. This should be discussed openly with families as part of shared decision-making [1,15].

Given the high placebo response and the limited evidence base for most agents, preventive therapy is best approached as an individualized, time-limited therapeutic trial (minimum two months, as supported by the 2019 AAN/AHS guideline) with explicit pre-treatment documentation of headache frequency, severity, disability, and acute-medication use, followed by structured reassessment of benefit, tolerability, and adherence before escalating or switching therapy [1,15]. No agent should be presented as universally superior or as a guaranteed first-line treatment. The magnitude and certainty of benefit vary across agents, and the placebo response in pediatric prevention trials is substantial. Treatment should be individualized according to disability, comorbidities, safety, adverse-effect profile, feasibility, and family preference [15]. Because pediatric migraine can change substantially with puberty and continued neurodevelopment, and because the disorder may itself improve with age, a preventive treatment that is working well should not be assumed to be needed indefinitely; periodic reassessment, with a trial of tapering or discontinuation once headache has been well controlled for a sustained period, is a reasonable and pediatric-specific consideration that the trial evidence reviewed above does not itself directly address.

Emerging therapies

Since 2024, several mechanistically novel therapies have entered pediatric migraine practice or investigation, with evidence and regulatory status varying substantially by agent [17]. Fremanezumab, a CGRP monoclonal antibody, now has phase 3 randomized pediatric efficacy evidence and is FDA-approved for episodic migraine in patients aged 6-17 years weighing at least 45 kg, the only FDA pediatric indication currently established among CGRP monoclonal antibodies [18,19]; other agents in this class remain at earlier pediatric pharmacokinetic/safety or investigational stages. The oral gepants rimegepant and atogepant have pediatric pharmacokinetic/safety and retrospective real-world data but no completed pediatric efficacy trial [20]. Remote electrical neuromodulation is FDA-cleared for acute and preventive treatment of migraine from age eight years, supported by pediatric real-world effectiveness data [21,22]. Pituitary adenylate cyclase-activating polypeptide (PACAP)-targeted therapy remains an adult-only, investigational mechanistic target with no pediatric data [23]. For most children today, these newer options are best regarded as second-line or adjunctive: only fremanezumab currently has trial-level pediatric evidence and a pediatric regulatory label, and the others are reasonable to consider once the established preventive measures described above have failed, are not tolerated, or are otherwise inappropriate, rather than as an initial choice. Table 3 brings together acute, preventive, and emerging pharmacological options for pediatric migraine, with typical pediatric dosing, evidence, and key cautions for each.

**Table 3:** Acute and preventive pharmacological treatment of pediatric migraine. Evidence sources are cited per agent within the table Source: [5,14,15,17-25] GI - gastrointestinal; NSAID - nonsteroidal anti-inflammatory drug; RCT - randomized controlled trial; SC - subcutaneous; CGRP - calcitonin gene-related peptide; PACAP - pituitary adenylate cyclase-activating polypeptide; CBT - cognitive-behavioral therapy; FDA - US Food and Drug Administration.

Drug/intervention	Pediatric age/population	Dose/regimen	Evidence/use	Key cautions
Acute treatment				
Ibuprofen	Children/adolescents	7.5-10 mg/kg	First-line; strongest evidence [5]	GI upset
Acetaminophen/paracetamol	Children/adolescents	15 mg/kg	Alternative first-line [5]	Hepatotoxicity in overdose
Sumatriptan/naproxen	≥12 years	10/60, 30/180, or 85/500 mg	Most established triptan combination [5,14]	Triptan contraindications
Zolmitriptan / sumatriptan nasal spray	≥12 years	5 mg / 20 mg	Useful when nausea limits oral therapy [5]	Triptan contraindications
Rizatriptan orally disintegrating tablet	≥6 years	5 mg (<40 kg) or 10 mg (≥40 kg)	Broadest pediatric approval [5]	Triptan contraindications
Almotriptan	≥12 years	6.25 or 12.5 mg	Guideline-recommended [5]	Triptan contraindications
Preventive treatment				
Topiramate	≥12 years	2-3 mg/kg/d (max ~200 mg/d)	Probable benefit vs placebo [15,24]	Cognitive slowing; teratogenic [15]
Propranolol	No contraindication	~20-40 mg three times daily (varies)	Possible benefit; low-confidence evidence [15]	Avoid in asthma/heart block/depression [15]
Amitriptyline + CBT	Migraine-related disability	0.25-1 mg/kg/d	Best evidence with CBT; monotherapy unproven [15]	Sedation; boxed suicidality warning [15]
Divalproex/valproate	Not routine first-line	15-30 mg/kg/d (up to 1000 mg/d)	Insufficient evidence vs placebo [15]	Teratogenic; avoid in reproductive age [15]
Cinnarizine	Not available in US/Canada	1.5 mg/kg/d (<30 kg) or 50 mg/d (>30 kg)	Probable benefit; single trial + meta-analysis [15,24]	Sedation; limited availability [15]
OnabotulinumtoxinA	Chronic migraine, specialist only	74-155 units intramuscular	Not superior to placebo in pediatric RCT [15,25]	Not established for routine use [15]
Emerging/advanced therapies				
Fremanezumab (CGRP monoclonal antibody)	6-17 years, ≥45 kg	225 mg SC monthly	Positive phase 3 RCT [17,18,19]	Injection-site reactions
Gepants (rimegepant, atogepant)	No completed pediatric RCT	Not established	Real-world benefit signal (Shin et al., 2026, multicenter chart review) [20]	Insufficient pediatric efficacy evidence
Remote electrical neuromodulation (REN)	≥8 years	Wearable device	FDA-cleared for acute and preventive migraine treatment from age 8 years; pediatric real-world evidence [21,22]	Device cost/access
PACAP-targeted therapy	Adult-only	Not applicable	Investigational [23]	Not for pediatric use

Antiemetics may be added according to age- and weight-appropriate local pediatric protocols when nausea or vomiting is prominent. For emerging and advanced therapies, evidence and regulatory status vary substantially by agent: only fremanezumab currently has trial-level pediatric efficacy evidence and a pediatric regulatory label, and pediatric long-term safety and comparative-effectiveness data remain limited for the other agents in this category.

Management of tension-type headache

TTH management is tiered: lifestyle measures first, behavioral therapy (CBT, relaxation, biofeedback) second, and pharmacological treatment - paracetamol or ibuprofen for acute attacks, and low-dose amitriptyline sometimes used for prophylaxis in refractory cases - reserved for children not responding to non-pharmacological measures, with the caveat that pediatric-specific prophylactic evidence remains very limited and use is off-label [7].

Management of cluster headache and other trigeminal autonomic cephalalgias

Because pediatric-specific controlled trial data for cluster-headache treatment do not exist, treatment is generally divided, as in adults, into acute (abortive), preventive, and transitional (bridging) therapy, with pediatric dosing derived from case series and adult practice rather than randomized pediatric trials (Table 4) [3,4].

**Table 4:** Pediatric trigeminal autonomic cephalalgias: clinical features and management Source: [3,4] TAC - trigeminal autonomic cephalalgia; SUNCT - short-lasting unilateral neuralgiform headache attacks with conjunctival injection and tearing; SUNA - short-lasting unilateral neuralgiform headache attacks with cranial autonomic symptoms; ECG - electrocardiogram

TAC	Typical attacks	Key distinguishing feature	Management
Cluster headache	15 min-180 min; one every other day to 8/day, children often 1-6/day	Unilateral pain with ipsilateral autonomic signs	Acute: 100% oxygen 7-12 L/min via non-rebreather mask; intranasal or subcutaneous sumatriptan/zolmitriptan in selected cases. Preventive: verapamil is commonly used, reflecting evidence largely extrapolated from adults and supported mainly by pediatric case-series experience; specialist supervision and ECG monitoring are required. Alternatives include gabapentin, topiramate, and melatonin; oral prednisone may be used as short transitional therapy [3]
Paroxysmal hemicrania	2 min-30 min; >5/day	Dramatic, near-diagnostic response to indomethacin	Indomethacin is both diagnostic and therapeutic
SUNCT/SUNA	5s-240s; 3-200/day	Very brief, very frequent attacks with cranial autonomic signs	Lamotrigine, topiramate, or gabapentin have been used, reflecting evidence largely extrapolated from adults and limited pediatric experience; pediatric evidence is insufficient, based only on case reports
Hemicrania continua	Continuous with exacerbations	Absolute response to indomethacin (required for diagnosis)	Indomethacin

Pediatric TAC treatment rests on evidence largely extrapolated from adults and should be specialist-guided (Table 4) [3,4]. Correct phenotype recognition carries direct treatment implications, given inconsistent pediatric use of the most effective acute therapies; a therapeutic indomethacin trial is reasonable in any child with a compatible unilateral headache phenotype once secondary causes have been excluded [1,3].

Medication-overuse headache

Medication-overuse headache should be considered in any child or adolescent with frequent or chronic headache and regular use of acute analgesics or triptans. ICHD-3 defines medication-overuse headache by headache on 15 or more days per month together with regular overuse of acute medication for more than three months, with an overuse threshold of 15 or more treatment-days per month for simple analgesics and 10 or more days per month for triptans and combination analgesics [2]; the AAN/AHS acute-treatment guideline's practical counseling advice of 14 and nine days respectively is intended to keep patients below these diagnostic thresholds, not to define an acceptable level of routine use [5]. Medication overuse appears to be less common in adolescents than in adults, plausibly because younger patients depend on caregivers for access to medication, but a careful medication-exposure history (including over-the-counter use) should be taken in every child with frequent headache [7]. Management principles are to establish the diagnosis, counsel the family on the relationship between medication frequency and headache chronification, taper or discontinue the overused agent under appropriate supervision, and initiate an evidence-based preventive strategy; anticipatory counseling at the time acute treatment is first prescribed is the most effective preventive measure [1,5].

Practical clinical approach

Figure 1 summarizes this approach as a stepwise algorithm; in narrative form, it proceeds as follows. A child presenting with headache should first undergo a detailed history (Table 1) and a general and neurological examination. If red flags or an abnormal neurological finding are present (Table 2), targeted investigation directed at the specific concern should follow, with treatment of the underlying cause and reassessment of headache management once the secondary cause is addressed or excluded. If absent, the primary headache phenotype should be identified - migraine, tension-type headache, a trigeminal autonomic cephalalgia, or another primary headache (Table 1) - and disability, frequency, and acute-medication use assessed (PedMIDAS or equivalent). Acute management and lifestyle/behavioral measures appropriate to the phenotype should be initiated, and the need for preventive treatment assessed: frequent, prolonged, or disabling headache, or inadequate, poorly tolerated, or overused acute treatment. If preventive treatment is indicated, an evidence-based agent individualized to comorbidities and adverse-effect profile should be selected and initiated as a time-limited therapeutic trial. Follow-up should reassess headache frequency, disability, treatment response, and adverse effects at a defined interval, using a headache diary, with the diagnosis reconsidered in non-responders before treatment is escalated.

**Figure 1:**
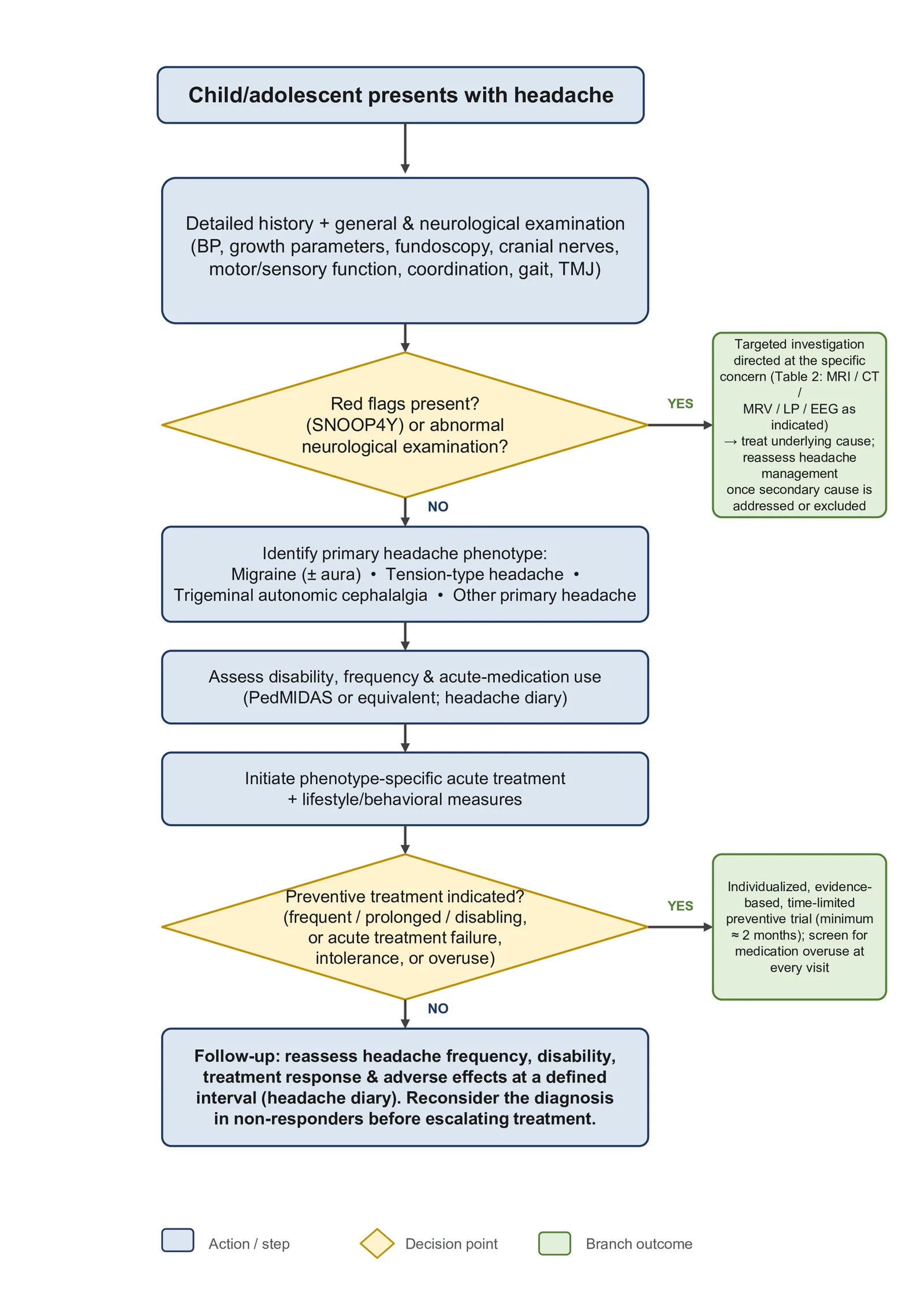
Practical approach to the child presenting with headache Original figure created by the author. BP - blood pressure; TMJ - temporomandibular joint; SNOOP4Y - Systemic signs/symptoms, Neurologic signs/symptoms, Onset sudden, Onset in sleep/early morning, Positional exacerbation/Precipitated by Valsalva, (lack of family history in) Parents, Progressive or new, and Young age; MRV - magnetic resonance venography; LP - lumbar puncture; EEG - electroencephalography; PedMIDAS - Pediatric Migraine Disability Assessment

The purpose of this approach is not to rule out everything with investigation, but to establish the most likely specific diagnosis, identify the children whose presentation genuinely warrants investigation, and match treatment intensity to headache burden and disability. The algorithm in Figure 1 is intended as a decision aid to support this reasoning, not as a rigid protocol to be applied without clinical judgement.

## Conclusions

Pediatric headache is best approached through structured clinical phenotyping, careful assessment for red flags, measurement of disability, and treatment tailored to the child's headache burden and phenotype. Most children with a typical primary headache phenotype and normal neurological examination do not require routine investigation. Management should combine education and lifestyle measures with appropriate acute treatment, while preventive therapy should be individualized according to disability, treatment response, comorbidities, and family preferences. Regular reassessment is essential, particularly when treatment response is inadequate or when the clinical phenotype changes.
